# Simulation and evaluation of increased imaging service capacity at the MRI department using reduced coil-setting times

**DOI:** 10.1371/journal.pone.0288546

**Published:** 2023-07-27

**Authors:** Ying-Chou Sun, Hsiu-Mei Wu, Wan-You Guo, Yang-Yu Ou, Ming-Jong Yao, Li-Hui Lee

**Affiliations:** 1 Department of Radiology, Taipei Veterans General Hospital, Taipei, Taiwan; 2 School of Medicine, National Yang Ming Chiao Tung University, Taipei, Taiwan; 3 Department of Medical Imaging and Radiological Technology, Yuanpei University of Medical Technology, Hsinchu, Taiwan; 4 Department of Health Care Management, National Taipei University of Nursing and Health Sciences, Taipei, Taiwan; 5 Department of Transportation and Logistics Management, National Yang-Ming Chiao Tung University, Hsinchu, Taiwan; First Technical University, NIGERIA

## Abstract

The wait times for patients from their appointments to receiving magnetic resonance imaging (MRI) are usually long. To reduce this wait time, the present study proposed that service time wastage could be reduced by adjusting MRI examination scheduling by prioritizing patients who require examinations involving the same type of coil. This approach can reduce patient wait times and thereby maximize MRI departments’ service times. To simulate an MRI department’s action workflow, 2,447 MRI examination logs containing the deidentified information of patients and radiation technologists from the MRI department of a medical center were used, and a hybrid simulation model that combined discrete-event and agent-based simulations was developed. The experiment was conducted in two stages. In the first stage, the service time was increased by adjusting the examination schedule and thereby reducing the number of coil changes. In the second stage, the maximum number of additional patients that could be examined daily was determined. The average number of coil changes per day for the four MRI scanners of the aforementioned medical center was reduced by approximately 27. Thus, the MRI department gained 97.17 min/d, which enabled them to examine three additional patients per month. Consequently, the net monthly income of the hospital increased from US$17,067 to US$30,196, and the patient wait times for MRI examinations requiring the use of flexible torso and head, shoulder, 8-inch head, and torso MRI coils were shortened by 6 d and 23 h, 2 d and 15 h, 2 d and 9 h, and 16 h, respectively. Adjusting MRI examination scheduling by prioritizing patients that require the use of the same coil could reduce the coil-setting time, increase the daily number of patients who are examined, increase the net income of the MRI department, and shorten patient wait times for MRI examinations. Minimizing the operating times of specific examinations to maximize the number of services provided per day does not require additional personnel or resources. The results of the experimental simulations can be used as a reference by radiology department managers designing scheduling rules for examination appointments.

## Introduction

MRI examinations are crucial in both clinical diagnosis and treatment as well as medical imaging research [1–3]. However, the wait time for patients from an appointment to a magnetic resonance imaging (MRI) service is usually long, with wait times generally ranging from 65 to 105 days [4, 5]. To achieve a higher scanner utilization rate and shorten wait times, radiology departments can either invest more in their workforce and facilities or adapt their existing services to enable the provision of more services. Both methods can be used to mitigate workflow bottlenecks. A department investing in its workforce and facilities can enhance the service levels of workflows that yield a low performance [6–8], and adapting existing services can enable a department’s resources and services to be rearranged to maximize resource performance [9]. Managers are more likely to adapt existing services than to increase their investment in their workforce and facilities. However, few studies have investigated the methods managers employ for service adaptation and the methods’ effectiveness.

A study showed that arranging MRI examinations according to examination types could shorten patients’ wait times since less room preparation is needed for the same service [9]. However, the same or different MRI examination types could require the use of different types of coils. For example, a brain MRI examination requires the use and preparation of an 8-inch head coil or standard head coil. An abdomen MRI examination needs the use and preparation of a torso coil. Therefore, MRI appointments may need to be scheduled by the coil type required for the examination instead of the examination type. Typical approaches to comparing and evaluating proposed solutions include the Lean philosophy [10], Lean Six Sigma [11, 12], plan–do–study–act [13], and simulations [14, 15]. Simulations are particularly advantageous because they can be employed without additional investment in a department’s workforce or physical resources being required. In addition, studies have verified the cause–effect relationship between input parameters and outputs [14, 15]. Most studies have used discrete-event simulation (DES) as a primary method for health service simulation [16, 17]. However, some complex health-care services cannot be adequately modeled using a single simulation method [18]. To model the behavior of patients interacting with each other and those competing for the same resources, agent-based simulation (ABS) is the best candidate method [18–21].

This study adopted a hybrid simulation model that combined DES and ABS to simulate MRI service operations in a radiology department in a medical center in northern Taiwan. To minimize adjustments to the medical center’s existing examination schedule, a solution was proposed wherein the examination room a patient was scheduled to undergo an examination in was determined on the basis of the coil type required for the examination. The following questions were explored:

1. To what extent does the proposed solution effectively reduce the number of MRI coil changes? Are fewer coil types used for each MRI scanner?
2. After the proposed solution is implemented, what is the maximum number of additional patients per day that can be scheduled for examinations?
3. How much does the monthly net income of the hospital increase? By how much can the wait time from scheduling an appointment to receiving an MRI be shortened?

## Literature review

### Simulation theory and methods

Simulation can be used in situations involving uncertainty and complex systems [19]. Simulation can also be used to test a proposed solution without the potential for equipment failure or business loss [22]. The output data obtained from a simulation analysis can be used to determine relationships between variables as well as causality between variables [14, 15]. Simulation methods commonly used in the literature are DES, ABS, system dynamics simulation, and the Monte Carlo method. The simulation method that is selected is determined by the type of problem to be solved [19]. Studies on the improvement of wait times have mostly used DES and ABS.

DES can model instantaneous changes in the operation of a system at discrete time points. It is mainly used to simulate the workflow of a system over time and is most commonly implemented for health-care simulations. DES is based on the queuing concept. Although DES incorporates time, it does not simulate interactions between individuals and does not account for the availability of resources, such as scanners [18–21]. The focus of ABS is the interactions between individuals, i.e., agents. Any entity that may compete with other entities can be considered an agent. Agents in the health-care domain include patients, physicians, nurses, and devices or scanners used in services or examinations. Each agent is independent and affects the other agents. ABS has the shortcoming that it is not based on the queuing concept [18, 19, 21]. Therefore, a combination of DES and ABS may be a suitable approach for simulating complex health-care situations to evaluate potential solutions.

### Application of simulation to improve patient wait times

Typical approaches employed to compare and evaluate proposed solutions to long wait times in the health-care domain include the Lean philosophy, Lean Six Sigma, plan–do–study–act, and simulations. The Lean philosophy, Lean Six Sigma, and plan–do–study–act require actual changes or investment in human and related resources. By contrast, simulations allow for the modeling of real-world environments and systems through the use of software, which enables a proposed solution to be implemented in a simulated model without affecting the current situation and without investment in resources. According to existing simulation-based evaluation studies, three strategies can be applied to shorten long wait times and increase the number of MRI department services that are provided: investing in additional workforce and related resources [7, 23–25]; minimizing the idle time of available scanners, rooms, and physicians involved in MRI examinations [8, 19, 26]; and minimizing the operation time of current services [9].

Regarding the first strategy of investment in additional workforce and related resources, a study employed DES and ABS to determine whether the number of MRI examinations conducted could be increased by adding the services of one radiation technologist during lunch period. The results revealed that 248 more patients received services each month. In addition, the results showed a 2.51-day reduction in wait times and a 6.15% increase in MRI scanner utilization [7]. Another study conducted a DES of the addition of one dosimetrist and reported a 6.55% reduction in the average wait time for radiation treatment in a radiation oncology clinic and an increase in the proportion of patients (84.92%) who would receive treatment within 14 working days. In another DES study, the addition of one MRI scanner and one radiation technologist at a military hospital in Iran resulted in an improvement in the medical personnel’s productivity and the elimination of patient wait time for turn-taking until admission [23]. In a Canadian study that employed a DES model, increasing the number of physician visits and sleep therapists reduced the wait times for examinations in a sleep therapy center by approximately 28 days [24]. Another DES study revealed that the addition of three surgeons and two physicians in an academic health science center in the United Kingdom shortened the wait time for bariatric surgery and related medical examinations (>18 weeks) in a bariatric center by 30% [25].

The second strategy for shortening wait times and increasing the number of MRI services provided, namely minimizing the idle time of available scanners, rooms, and physicians, does not require investment in additional human and related resources. A study in the United States investigated whether the wait times from consultation appointments to surgical evaluation and confirmation and finally to the scheduling of surgery could be shortened by redefining appointment rules to allow available surgeons to provide surgical evaluations and confirmations and thereby increasing the number of time slots available for appointments. The results revealed that wait times could be reduced by up to 70% and that three additional surgeries could be performed each month [8]. In addition, to reduce the wait times for radiotherapy from referral to the start of treatment, the Netherlands Cancer Institute developed a push and pull strategy for making appointments to reduce the time gap between the aforementioned processes. The pull aspect of the strategy involved first booking a radiotherapy appointment and then estimating the time that would be required to verify the tumor contour and location through imaging. The push aspect of the strategy involved booking a radiotherapy appointment only after a patient underwent imaging. The study employed DES and demonstrated that the use of push and pull strategies at a ratio of 40%:60% could reduce the average wait times for radiotherapy by 12% and the number of patients waiting by 41% [26].

The third strategy, which also does not require investment in additional human and related resources, involves minimizing the operation time of current services to increase the number of services that can be provided. However, studies on this strategy are lacking. In a Portuguese medical institution, DES was used to simulate the workflow of a radiology department. The results revealed that the reason for long patient wait times was radiology assistants having a heavy workload. The assistants were required to schedule patients for examinations and provide them with information as well as to assist in all radiological examinations, including computed tomography and ultrasound examinations. Because of their considerable workload, the assistants were often unable to notify patients of examinations in a timely manner. To solve this problem, the assistants’ workload was reduced, and the radiologists performed the radiological examinations alone, which resulted in a 41% reduction in examination time and wait time was decreased to 4.8 minutes [9]. Another study used simulated annealing algorithm to optimize the patient admission sequence at an imaging clinic. The results showed average reductions of 5% on the total completion time and 38% on the patients’ total waiting time when the MRI patients were admitted by examination type [9].

## Materials and methods

### MRI examination flow

**Fig 1:**
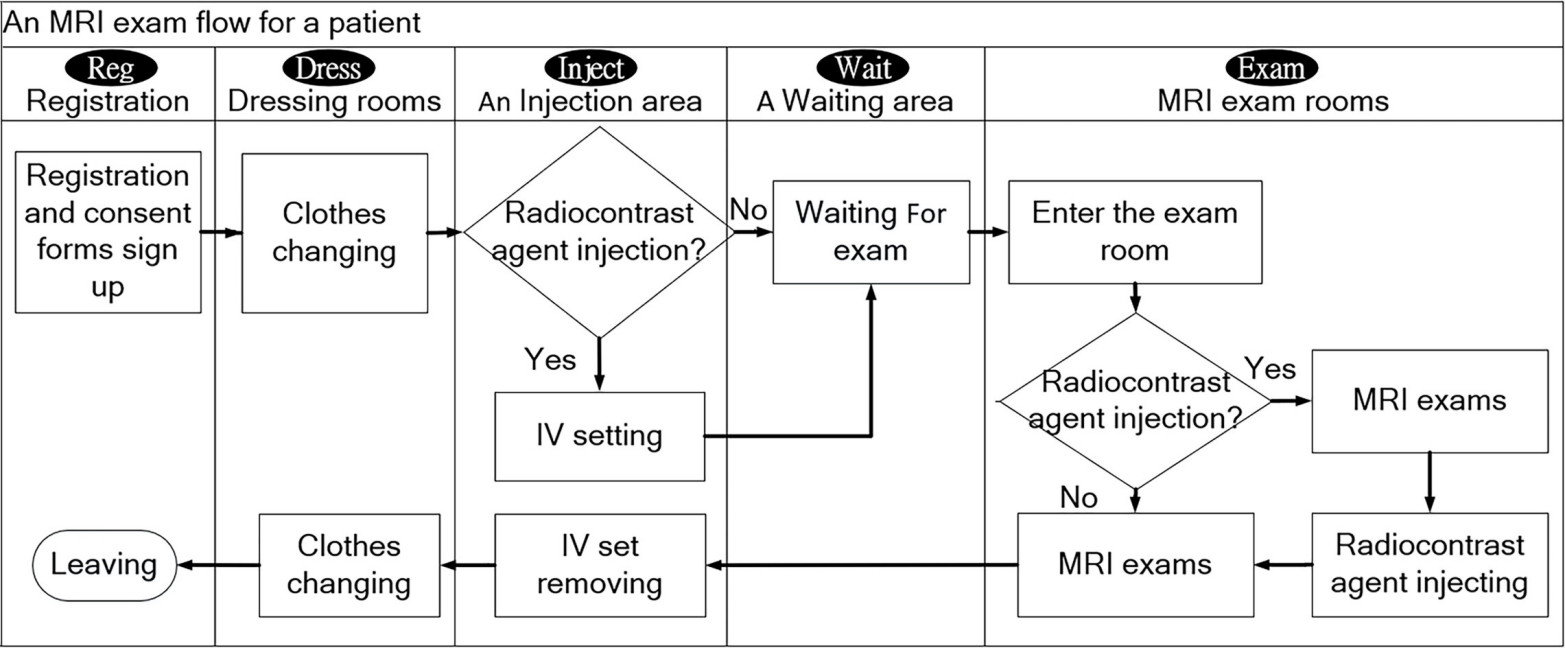
Flow of a patient MRI examination.

Fig 1 presents the flow of MRI examinations in Taipei Veterans General Hospital. The figure presents the operations implemented in five locations within the hospital. First, patients check in at the registration counter (Reg) and sign the consent form for MRI examinations. The patients then walk to the dressing room (Dress) to change into loose-fitting hospital clothing. If the patients require a radiocontrast agent injection (Inject), a nurse sets up intravenous (IV) equipment. Thereafter, the patients wait in a waiting area (Wait) until a radiation technologist calls them into the MRI examination room. The patients may undergo an examination with or without a radiocontrast agent injection (Exam). If a patient receives a radiocontrast agent injection (Inject), the inserted IV catheter is removed. Finally, the patients change back into their clothes (Dress) before leaving (Reg).

### Material and data preprocessing

A total of 2,447 MRI examination logs from December 2016 with deidentified patient data and information regarding the radiation technologist who completed the examination were collected from the MRI department. Because the examination schedule was fixed, more recent data sets were not required for the study analysis. Patient data, including sex, the type of medical service received (i.e., outpatient, emergency, or inpatient care), the need for injection of a radiocontrast agent, the type of MRI scanner used in the examination (labeled A to D), the type of MRI examination (51 recorded classifications), and the date and time of getting on and off the MRI examination table, were recorded. Other information, such as the department workflow, business hours, human resource allocation, and location of 1.5-T scanner, was collected using the method of authors Sun, Wu, and Guo. The present study was approved by the Institutional Review Board of Taipei Veterans General Hospital (Number: 2018-01-010CC). The need for consent was waived by the ethics committee (IRB).

Data preprocessing was conducted to generate input parameters, output validation indicators, and key performance indicators. Data regarding the 51 types of MRI examination were available in logs, with the data indicating that 11 types of coils were used. This study used WaitExamDay to represent the length of a patient’s waiting time, with the length calculated as the date and time (in h) at which an MRI examination was performed minus the date and time at which an examination was scheduled. DailyMRIexamTime refers to the date and time at which the scan was completed minus the date and time at which the patient got on the MRI examination table. For records with missing data on the date and time at which a patient got on or off the MRI examination table, DailyMRIexamTime was replaced with the mean DailyMRIexamTime. DailyOverTime represents the difference between the time at which a patient got off the MRI examination table and 23:00, which is the time at which service operations end each day.

### Descriptive statistics of source material

The average DailyMRIexamTime was 36 min (standard deviation [SD] = 2.04). The average number of daily coil changes (DailyCoilChangeOccurence) was 46.35 (SD = 6.05). The average MRI examination preparation time (MRIexamPrepTime) was 3.77 min (SD = 0.52). The average daily rates of utilization of the four MRI scanners available in the hospital (DailyScanUtilRate) were 85.06%, 85.48%, 85.20%, and 80.23%. The average DailyOverTime was 37.48 min (SD = 0.52).

The mean WaitExamDay values and median DailyMRIexamTime values for the MRI examinations involving each type of coil are listed in Table 1. The longest WaitExamDay (22 days) was for MRI examinations involving knee and shoulder coils, followed by that (21 days) for MRI examinations involving a flexible torso and head coil. The shortest WaitExamDay (0.96 days) was for examinations involving a standard head coil. The first and second longest DailyMRIexamTime (99 and 46 min, respectively) were for examinations involving cardiac and breast coils, respectively. The shortest DailyMRIexamTime (25 min) was for examinations involving spinal and lower extremity coils.

**Table 1:** Wait times for MRI examinations involving each coil type.

Coil types (abbreviation)	WaitExamDay	DailyMRIexamTime
	Mean (day)	Median (min)
Standard head (StdHead)	0.96 (23 hours)	36
Cardiac (Card.)	6	99
8-inch head (8chHead)	7	28
Neurovascular (Nuro.)	8	30
Breast	9	46
Torso	10	36
Spine	13	25
Low extremity (LowExtre)	15	25
Flexible torso and head (TorsoHead)	21	40
Knee	22	27
Shoulder	22	34

### Simulation models for MRI examinations

#### Simulation framework

**Fig 2:**
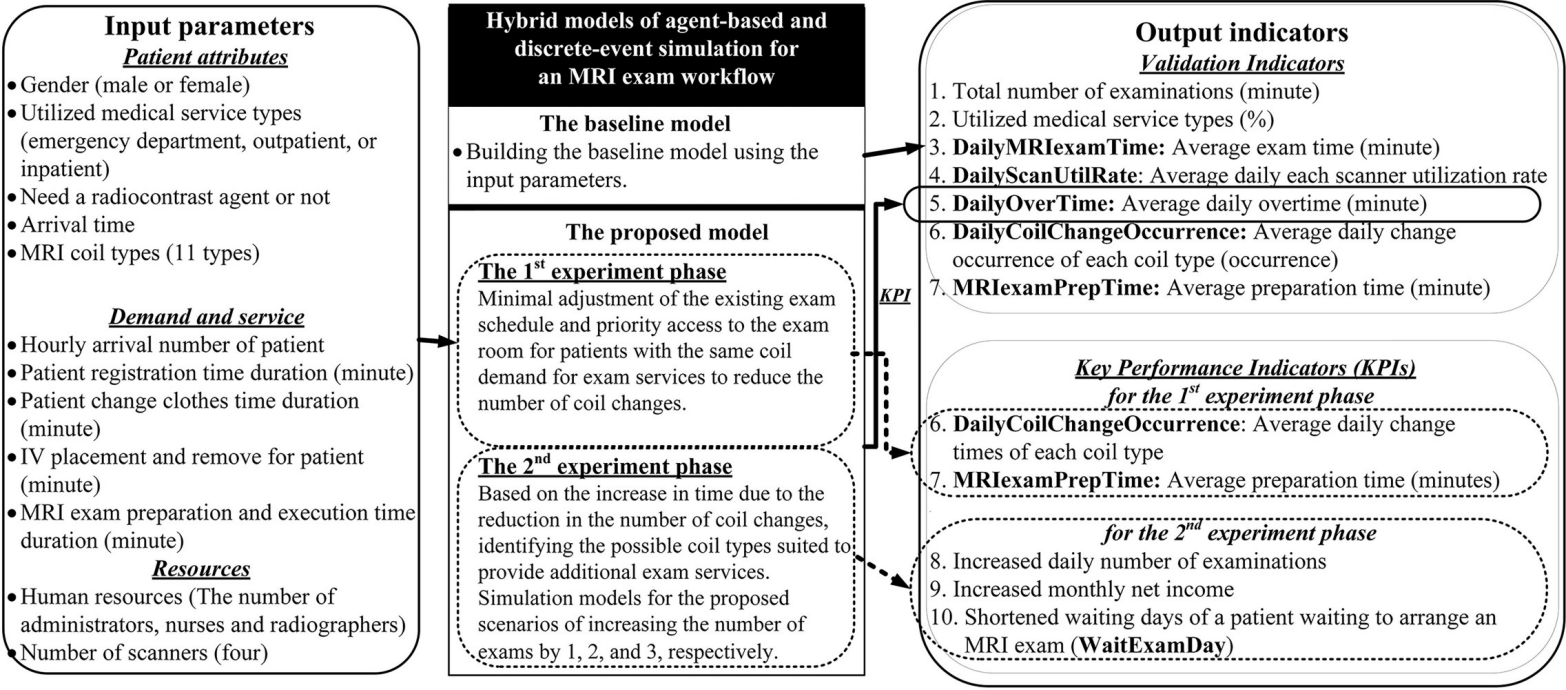
Simulation architecture.

The simulation framework (Fig 2) combined DES and ABS, which have been proposed by other studies [7, 27]. The input parameters included patient attributes, demand and services, and resources. Two simulation models were developed: a baseline model and a proposed model. The baseline model simulated the current MRI examination process, and for the proposed model, the baseline model was used as a prototype and the proposed scheme was implemented.

The validation indicators for the baseline model included the total number of examinations, the percentages of the types of medical services provided, DailyMRIexamTime, DailyScanUtilRate, DailyOverTime, DailyCoilChangeOccurrence, and MRIexamPrepTime. In addition to the validation indicators, the proposed model included key performance indicators that were analyzed in two experimental phases. The first experimental phase involved the DailyOverTime, DailyCoilChangeOccurence, and MRIexamPrepTime. The second experimental phase involved the DailyOverTime, increased daily examinations, increased monthly net income, and shortened WaitExamDay. The simulation period was December 1–31, 2016. For the collected validation indicators, the number of replications made after each run was eight at a 95% confidence level with one coil change.

#### Baseline model

An MRI examination simulation model was developed by combining DES and ABS.

**Fig 3:**
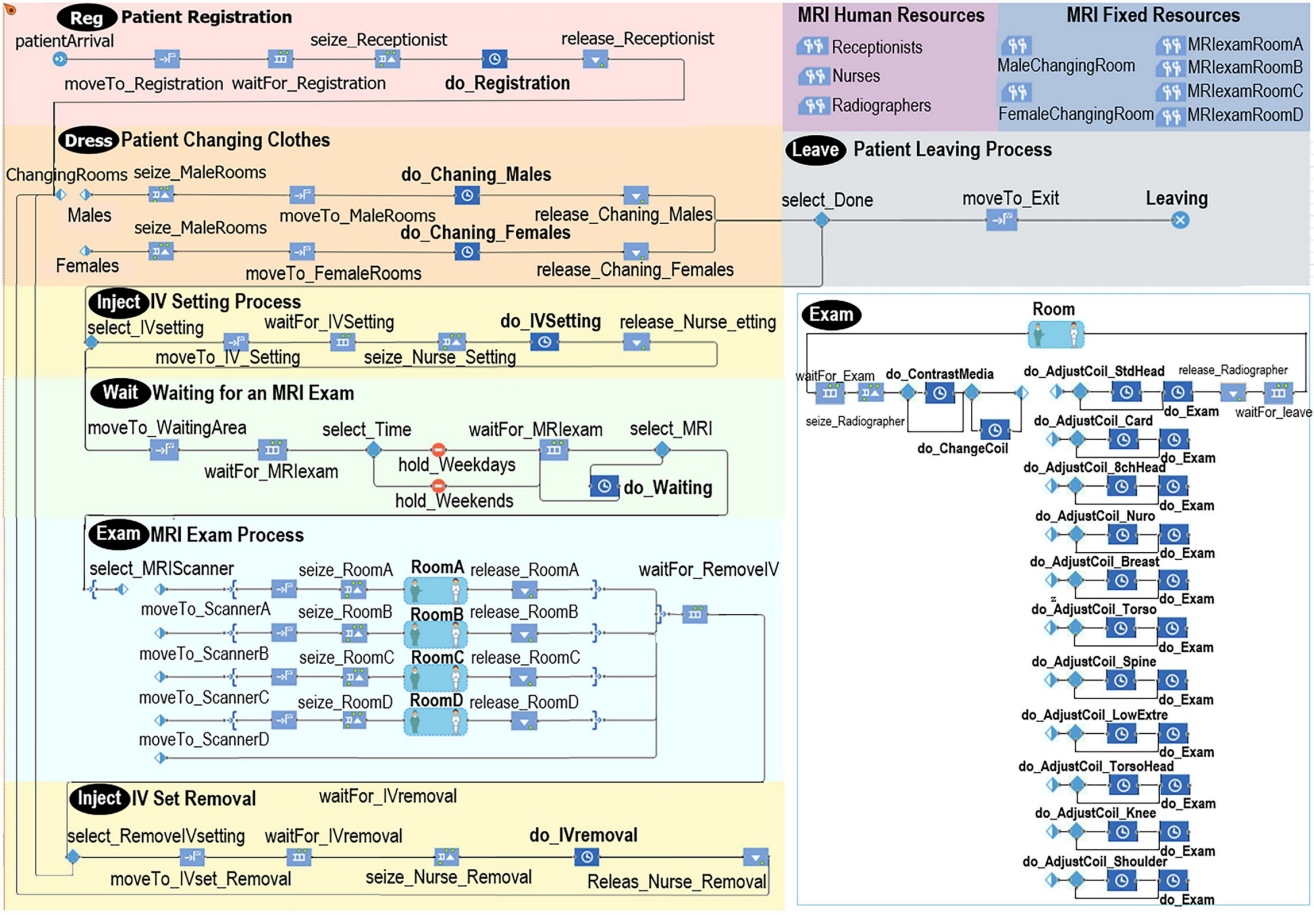
Baseline simulation model for MRI examinations.

*(1) DES for the MRI examination workflow*. The models (Fig 3) were designed on the basis of the MRI examination workflow (Fig 1) and the simulation framework (Fig 2). For the Patient Registration variable, the patient arrival time was determined by the number of patients per hour. For the workflow variable Patients Changing Clothes, patients entered different changing rooms according to their gender. The IV Setting indicated that the patient was given an injected with a contrast agent and was used to determine whether the nurse practitioner performed an IV catheterization. The examination room was selected for MRI Exam based on currently unused MRI scanners and the coil type that the given scanner could use. After the patient entered the room, a radiation technologist was responsible for the examination. The radiation technologist pre-adjusted the scanner depending on whether the patient required coil replacement. Finally, the scan was performed once or twice according to whether the patient needed a contrast agent injection. For IV Set Removal, the nurse removed the IV catheter. Once the examination was complete, the patient left the radiology department.

**Fig 4:**
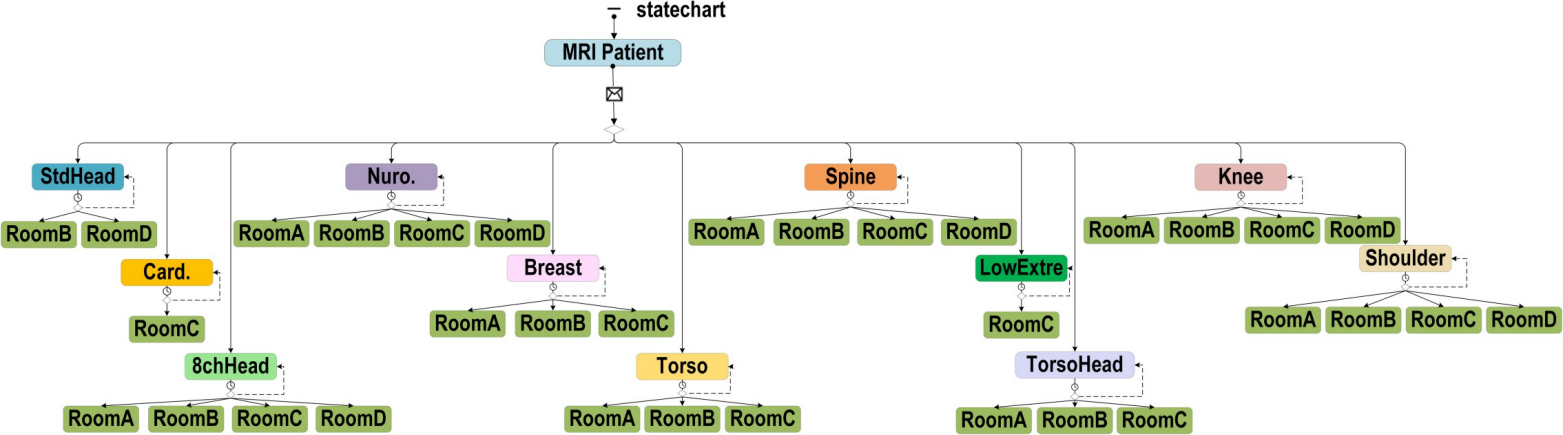
Baseline model of the use of MRI scanners for patient examinations.

*(2) ABS for the MRI examination workflow*. The ABS included agent patient attributes and changes in the availability status from different scanners. Patient attributes were set with actual occurrence ratios, including type, which we assigned to outpatient, emergency, and inpatient care; gender, which was either male or female; setupIV, which was whether the patient required an injection during the examination; and priority, which prioritized patients who were in an emergency condition. The current Time was the time at which the patient arrived at the radiology department. The dates were categorized by day of the week, and the time was divided into morning, afternoon, and evening sessions. Each scanner could only accommodate specific coil types (Fig 4); therefore, each scanner had a different utilization rate depending on the coil type required by the patient. For example, the cardiology coil type could only be used in Scanner C, i.e. Room C, and consequently had a 100% chance of being used. The standard head coil type could be used in Scanners C and D and had 3% and 97% chances of being used in each scanner.

#### Proposed model

**Fig 5:**
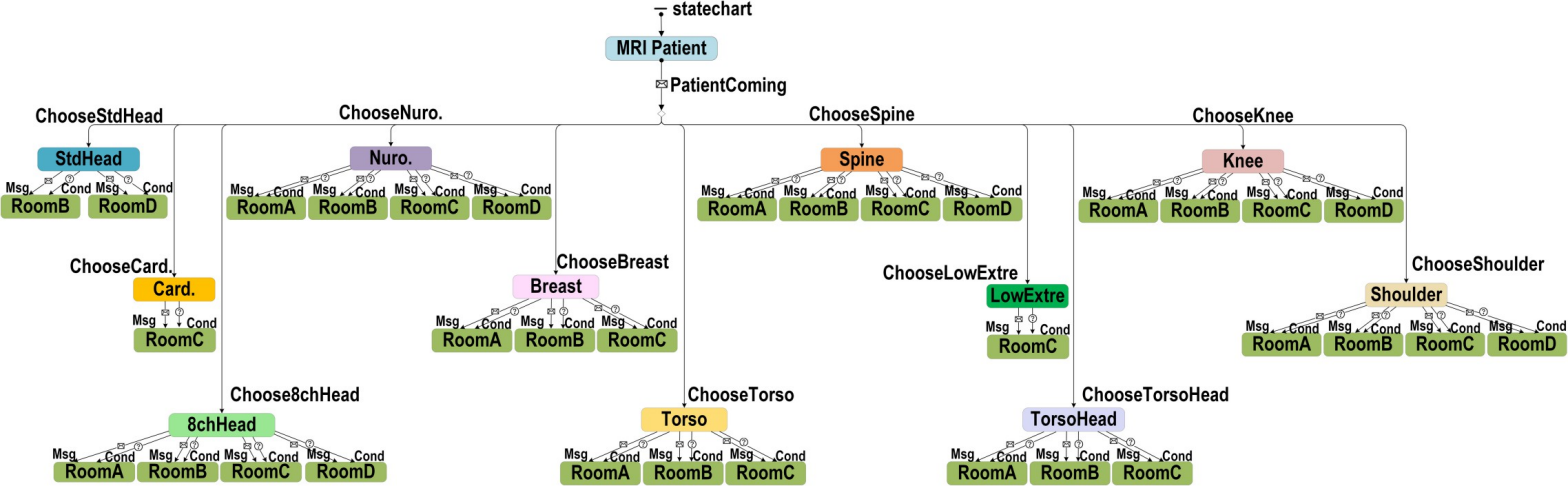
Proposed model of the use of MRI scanners for patient examinations.

Two experimental phases were established in the proposed model. In the first experimental phase, a week was divided into three groups: Sunday and Monday, Tuesday and Wednesday, and Thursday to Saturday. We reduced the number of coil changes by arranging the same coil types in each group to be in the same or adjacent time slots (Fig 5). The utilization rate of MRI scanners was also considered so that each scanner could be used at each time slot. The number of patients increased in the second experimental phase based on the results of the first experimental phase. The amount of time saved by reducing the number of coil changes was used as a criterion; the additional coil types and examination numbers per day were evaluated based on the median number of scans performed for each type of coil. One, two, and three additional patients a day were added to the experiment to evaluate the increase in the monthly net income and the reduction in the patient waiting time for MRI examinations for a given coil type.

### Model validation and tools

Data were preprocessed on Microsoft Office Excel 365. AnyLogic was used to construct the baseline and proposed simulation models. SAS 9.4 was used to evaluate the significant differences between the real data and the baseline model and between the baseline and proposed simulation models. The Wilcoxon rank-sum test was used because the samples were nonnormally distributed. A *p* value of <0.05 indicated statistical significance.

## Results

### Baseline model validation

As indicated in Table 2, the total number of examinations included in the baseline simulation model was 2,449. The majority of the sample was outpatients (1,817 patients; 74.18%), followed by inpatients (491; 20.06%) and emergency patients (141; 5.74%). The average examination time was 32.52 min. The average MRI examination preparation time was 5.27 min. The average daily utilization rates of Scanners A to D were 85.63%, 86.23%, 87.05%, and 80.23%, respectively. The average daily overtime was 40.23 min, and the average daily number of coil changes was 40.38. Nonsignificant differences were observed between the validation indicators of the baseline simulation model and real data, which indicated that the baseline simulation model was consistent with the actual situation.

**Table 2:** Results of the Wilcoxon rank-sum test of validation indicators between the baseline model and real data.

Validation indicators	Real Data		Baseline model		*p*-value
Total number of exams: n	2,447		2,449		0.6437
Utilized medical service types: n (%)					
Outpatient	1,819	(74.34)	1,817	(74.18)	0.6673
Emergency department	145	(5.93)	141	(5.74)	0.7992
Inpatient	483	(19.74)	491	(20.06)	0.4888
DailyMRIexamTime: mean (SD)	33.03	(1.97)	32.52	(1.07)	0.2975
MRIexamPrepTime: mean (SD)	5.07	(0.63)	5.27	(0.42)	0.1024
DailyScanUtilRate: %					
Scanner A	85.06		85.63		0.6322
Scanner B	85.48		86.23		0.5733
Scanner C	85.20		87.05		0.8769
Scanner D	81.30		80.23		0.0911
DailyOverTime: mean (SD)	37.48	(19.38)	40.23	(12.05)	0.2397
DailyCoilChangeOccurrence: mean (SD)	43.35	(6.05)	40.38	(5.79)	0.0950

### Proposed model validation

In the first experimental phase, minimal adjustments were made to the existing examination schedule, and scheduling for examination rooms was completed with consideration of whether patients required examinations involving the same coil type.

As indicated in Table 3, the average daily overtime reduced from 40.23 to 30.32 min after implementing the proposed scheme (*p* = 0.0013). The average number of coil changes per day for all scanners decreased from 40.38 to 13.04 times (*p* < 0.0001). Moreover, the total number of coil changes per day declined (Table 4). The average preparation time was reduced from 5.27 to 4.04 min (*p* < 0.0001), which reduced the total preparation time (i.e., the time required to change the coil) by 97.17 min.

**Table 3:** Results obtained in the first experimental phase of the proposed model (N = 2,499).

KPIs mean ± SD	The baseline model		The proposed model		*p-value*
DailyOverTime: minutes	40.23	+12.05	30.32	+9.03	0.0013^**^
DailyCoilChangeOccurrence: occurrence	40.38	+ 5.79	13.04	+3.54	<0.0001^***^
MRIexamPrepTime: minutes	5.27	+ 0.42	4.04	+0.29	<0.0001^***^

**p < 0.01

*** *p* < 0.001

**Table 4:** Number of coil changes for each MRI scanner.

Scanner (Available coil types) Coil change occurrences	Sun.	Mon.	Tue.	Wed.	Thur.	Fri.	Sat.
Scanner A (8)	13→4	14→5	11→5	12→3	12→4	15→3	11→4
Scanner B (9)	12→5	13→4	10→5	13→4	12→4	13→2	11→4
Scanner C (10)	13→5	14→5	13→5	15→3	12→3	12→3	11→4
Scanner D (7)	11→4	9→3	8→5	9→2	11→4	9→4	8→2

**Fig 6:**
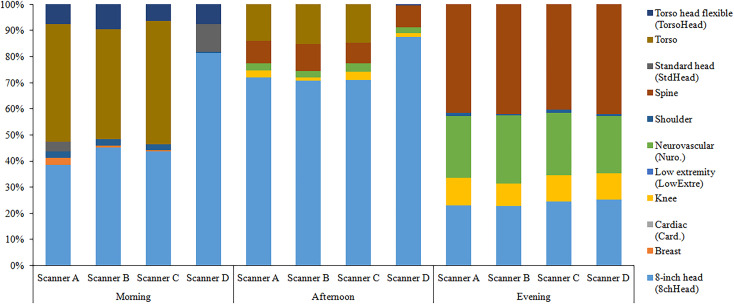
Proportion of coil type used for each MRI scanner in the session time in the proposed model.

After the implementation of the proposed model, the proportion of coil type used for each MRI scanner in the session time was calculated (Fig 6). The results revealed 8–15 coil changes for each MRI scanner in the session time occurred in the baseline model, whereas 2–6 changes that occurred for the same scanner and session in the proposed model.

The results of the second experimental phase are presented in Table 5. The time saved as a result of the reduced number of coil changes enabled the examination of three additional patients per day.

**Table 5:** Simulation results for the proposed model.

Experiments: Added No. of patients per day	Total No. of patients in the month	Increase No. of patients in the month (%)	The utilization rate of the MRI scanner (daily) (%, used hours / 16 hours)				Average daily overtime minutes	① Increased point of hospital global budget payment per month		② Increased net income per month (31 days【① x 0.8470 x 0.03US】	
			A	B	C	D		Without contrast^†^	With contrast^‡^	Without contrast	With contrast
**baseline**	**2,449**		**85.63**	**86.23**	**87.05**	**80.23**	**40**				
proposed	2,449		81.19	82.47	81.39	75.96	30				
+ 1	2,480	1.27	82.75	83.82	83.48	77.84	37	201,500	356,500	5,689	10,065
+ 2	2,511	2.53	83.58	84.57	84.21	79.37	40	403,000	713,000	11,378	20,130
**+ 3**	**2,542**	**3.80**	**84.25**	**85.14**	**84.76**	**80.59**	**43**	**604,500**	**1,069,500**	**17,067**	**30,196**

†Without contrast: number of monthly patients multiplied by the global points of payment of the hospital budget for providing each MRI examination to a patient without radiocontrast agent injection (6,500 points)

^‡^with contrast: number of monthly patients multiplied by the global points of payment of the hospital budget for providing each MRI examination to a patient with radiocontrast agent injection (11,500 points).

Completing one additional examination per day led to a 1.27% total increase in the number of examinations performed in a month, and the average utilization rate of the MRI scanners increased by 1.72%. The payment points of the monthly global hospital budget for MRI examinations with and without radiocontrast agent injection increased to 356,500 and 201,500, respectively. Thus, the net income of the hospital increased to US$10,065 and US$5,689 for MRI examinations with and without radiocontrast agent injection, respectively.

Completing two additional examinations per day led to a 2.53% total increase in the number of examinations conducted, and the average utilization rate of the MRI scanners increased by 2.68%. The payment points of the monthly global hospital budget for MRI examinations with and without radiocontrast agent injection increased to 713,000 and 403,000, respectively. Thus, the hospital’s net income increased to US$20,130 and US$11,378 for MRI examinations with and without radiocontrast agent injection, respectively.

Completion of three additional examinations per day led to a 3.80% total increase in the number of examinations conducted, and the average utilization rate of the MRI scanners increased by 3.43%. The payment points of the monthly global hospital budget for MRI examinations with and without radiocontrast agent injection increased to 1,069,500 and 604,500, respectively. Thus, the hospital’s net income increased to US$30,196 and US$17,067 for MRI examinations with and without radiocontrast agent injection, respectively.

The *p* values for overtime before and after the one, two, and three additional examinations were 0.1928, 0.8714, and 0.1530, respectively, indicating statistical nonsignificance. This study analyzed the utilization of flexible torso and head, shoulder, 8-inch head, and torso coils when three additional patients completed examinations each month and discovered that the wait times of the last patients on the waiting list for examinations involving these coils were shortened by 6 d and 23 h, 2 d and 15 h, 2 d and 9 h, and 16 h, respectively (Table 6).

**Table 6:** Reductions in patient wait time for MRI examinations involving four coil types when three additional patients are examined per day.

Add coil types			The baseline model		The proposed model	
	No. of exams	Average waiting time for MRI exam	Exam time duration (median)	The last exam date in the month	No. of exams	Shorten the waiting time of patients for MRI exam
TorsoHead	53	21 days	40 minutes	2016/12/30 17:40	77	6 days and 23 hours
Shoulder	41	22 days	34 minutes	2016/12/30 17:30	43	2 days and 15 hours
8chHead	398	7 days	28 minutes	2016/12/31 22:17	1,369	2 days and 9 hours
Torso	1,261	21 days	36 minutes	2016/12/31 21:35	405	16 hours

## Discussion

In the present study, problems often encountered in the examination process of MRI departments were investigated and a workflow that would shorten patient examination times was proposed. The average number of coil changes per day was reduced by approximately 27. Thus, a total of 97.17 min was saved, which resulted in an additional three patients being able to undergo examinations involving flexible torso and head, shoulder, 8-inch head, and torso coils each day. The average daily utilization rates of scanners A, B, C, and D increased by 3.06%, 2.26%, 3.37%, and 4.63%, respectively. The hospital’s monthly net income was determined to have potential to increase by US$17,067 to US$30,196. In addition, the wait time of the last patient on the wait list for an examination involving the aforementioned coils was reduced by 16 h to 6 d and 23 h. To achieve these results, the number of employed staff and scanners need not be increased.

This study discovered that reducing the number of coil changes shortened the MRI examination preparation time. This finding is consistent with the findings of other study, which reported that examination preparation time can be reduced by minimizing inefficient labor and work allocation [9]. The additional time that resulted from the reduced wait times can enable three additional monthly procedures to be performed [8]. Additionally, the increase in the number of available time slots would enable patients who are last on the waiting list to undergo an examination earlier [7]. The approach proposed in this study could reduce the number of unnecessary coil changes and shorten the examination preparation time to thereby reduce patients’ wait times.

A study has reported that patient wait times can be reduced through the adjustment of examination schedules and that flexible scheduling based on the examination length is more effective than using a fixed length of time for all examinations [28]. In this study, scheduling was based on the examination length as well. However, when a study has not considered whether the coils used for each examination were the same over consecutive periods, it therefore could not identify the amount of time wasted during MRI examinations.

Another similar study showed reductions in the patients’ total waiting time when the MRI patients were admitted by examination type [9]. In this study, the scheduled examinations were arranged consecutively according to the same coil type examination instead of the examination type, which could more accurately reduce the time for coil change. In addition, there were more scanners and examinations per day in this study setting, so ABS was used to prioritize the examination of patients with the same coil type needed to avoid the on-site patient wait times and staff overtime. Moreover, this study further simulated the financial income of additional examinations due to the increased time for service.

### Limitations and future work

This study has three main limitations. First, the raw data were incomplete; some parameters, such as the times of patient check-in, dressing, registration, and removal of IV settings, were unavailable. Therefore, the distribution of those parameters could only be set according to thumb rules. Moreover, several additional months of examination data are required to determine whether seasonal changes may occur.

Second, this study assumed that each scanner could be operated daily during business hours without regular or irregular maintenance. Thus, the utilization rates of the scanners may have been overestimated. Additionally, in this study, the walking speed was not separately set for older or less mobile patients because of the lack of raw data. In the future, a maintenance schedule for scanners should be included and the walking times of patients of different age and mobility groups should be estimated.

Third, the examination appointment was adjusted by including additional examinations involving the aforementioned coil types on the basis of the median time required for examinations of each type. However, because of the appointment rules of the research institution, only some examination schedules can be adjusted. In the present study, various combinations of coil and time slot arrangements were evaluated and the parameters were manually calculated and adjusted. The results reveal that the best method for scheduling may involve rearranging and adjusting the schedule on the basis of the monthly demand for examinations. In the future, a simulation analysis can be combined with machine learning strategies, such as reinforcement learning, to identify the optimal combination of MRI examinations for scheduling purposes.

## Conclusion and contribution

This study used hybrid simulations to analyze models, which enabled analyses to be conducted without investing actual human or material resources, which enabled this study to save on costs and avoid the consequences of failure. The use of a combination of DES and ABS enabled the simulation of changes in patient status because ABS can be used to transmit information to facilitate the identification of patients requiring examinations involving the same coils. This study identified potential areas in the current examination service flow in which time management could be improved. Notably, the number of coil types used and changes per day could be reduced by slightly adjusting the appointment time slots for MRI examinations involving each type of coil. The implementation of this strategy for minimizing the operating time of such examinations to increase the number of available services did not require additional human and related resources. The evaluation of the proposed strategy by using the hybrid simulation model revealed that after the implementation of the strategy, the available service times increased, the wait times for MRI examinations from appointments decreased, and the hospital’s net income increased. The results of this study can serve as a reference for radiology department managers for devising strategies for shortening lengthy wait times.

## Supporting information

S1 File (ZIP)
